# (3*β*,16*α*)-3,16-Dihydroxypregn-5-en-20-one from the Twigs of *Euonymus alatus* (Thunb.) Sieb. Exerts Anti-Inflammatory Effects in LPS-Stimulated RAW-264.7 Macrophages

**DOI:** 10.3390/molecules24213848

**Published:** 2019-10-25

**Authors:** Seulah Lee, Dahae Lee, Su Cheol Baek, Mun Seok Jo, Ki Sung Kang, Ki Hyun Kim

**Affiliations:** 1 School of Pharmacy, Sungkyunkwan University, Suwon 16419, Korea; sarahlee0801@gmail.com (S.L.); pjsldh@naver.com (D.L.); schii513@daum.net (S.C.B.); anstjr920827@gmail.com (M.S.J.); 2 College of Korean Medicine, Gachon University, Seongnam 13120, Korea

**Keywords:** *Euonymus alatus*, sterol, nitric oxide, inducible nitric oxide synthase, NF-κB

## Abstract

To discover new pharmacologically active natural products, here, we performed the phytochemical analysis of a Korean medicinal plant. *Euonymus alatus* (Thunb.) Sieb. is a traditional medicinal plant that has been used as a remedy for various diseases in Asian countries. In particular, the cork cambium on the twigs of *E. alatus* has been used to treat dysmenorrhea, tumors, diabetes, and wound. Phytochemical analysis of the methanolic extract of *E. alatus* twigs led to the isolation of a sterol, which was identified as (3*β*,16*α*)-3,16-dihydroxypregn-5-en-20-one (**1**) by 1D and 2D nuclear magnetic resonance (NMR) spectroscopy and high-resolution electrospray ionization mass spectrometry. The stereochemistry of **1** was established with nuclear Overhauser effect spectroscopy (NOESY) analysis and comparison of electronic circular dichroism (ECD) data. To the best of our knowledge, the isolation of compound **1** from nature is first reported here, as well as the complete and revised NMR data assignment of **1**. In lipopolysaccharide (LPS)-stimulated RAW-264.7 macrophages, compound **1** significantly inhibited nitric oxide (NO) production at an IC_50_ value of 12.54 ± 0.05 μM as well as the protein expression of inducible nitric oxide synthase (iNOS) and cyclooxygenase-2 (COX-2). Moreover, the pre-treatment with compound **1** attenuated the LPS-induced phosphorylation of nuclear factor kappa B (NF-κB) p65 through the inhibition of the phosphorylation of IκB kinase alpha (IKKα), IKKβ, and inhibitor of kappa B alpha (IκBα). Compound **1** also inhibited the LPS-induced phosphorylation of p38, c-Jun NH_2_-terminal kinase (JNK), and extracellular signal-regulated kinase (ERK). Taken together, compound **1** may serve as an anti-inflammatory constituent of *E. alatus* twigs and its anti-inflammatory property is thought to be associated with the inhibition of NO production via suppression of iNOS and COX-2 expression through inhibition of IKKα/β, I-κBα and NF-κB p65 activation and downregulation of p38, JNK, and ERK mitogen-activated protein kinase signal pathways in RAW 264.7 macrophages. These findings also provide experimental evidence that compound **1** identified from *E. alatus* twigs could be a candidate for an anti-inflammatory agent.

## 1. Introduction

Inflammation is a complex immune response of a host organism against bacteria, parasites, viruses, and fungi. During this process, pro-inflammatory cytokines such as the tumor necrosis factor (TNF-α), interleukin (IL)-6, and IL-1β as well as the endotoxins such as lipopolysaccharide (LPS) derived from the outer membrane of gram-negative bacteria are known to phosphorylate mitogen-activated protein kinases (MAPKs), including p38, c-Jun NH_2_-terminal kinase (JNK), and extracellular signal-regulated kinase (ERK). The consequence of this event includes the activation of nuclear factor kappa B (NF-κB) in macrophages [1,2]. LPS is also known to contribute to the expression of inducible nitric oxide synthase (iNOS) and cyclooxygenase-2 (COX-2), regulated by the transcription factor NF-κB via the Toll-like receptor (TLR) signaling pathway in monocytes/macrophages and neutrophils/granulocytes, and other immune cells [3,4]. The activation of iNOS leads to an increase in the level of nitric oxide (NO) [5], which plays a key role as a signaling molecule in the inflammatory response and has been implicated in the development of inflammation-associated diseases such as glomerulonephritis, vasculitis, arthritis, and asthma [6,7]. Thus, several natural products have been intensively investigated for their abilities to modulate the expression of iNOS, COX-2, NF-κB, and MAPKs (JNK, ERK, and p38) in RAW-264.7 macrophages [8,9,10,11].

*Euonymus alatus* (Thunb.) Sieb. (Celastraceae) is a deciduous and popular ornamental tree, commonly known as winged euonymus. This tree is also one of the well-known traditional medicinal plants that have been used to treat dysmenorrhea and prevent atherosclerosis development [12]. The cork cambium on the twigs of this plant, commonly known as ‘Gui-junwoo’ in Korean traditional medicine, has particularly been used for over 2000 years to regulate blood circulation, relieve pain, eliminate stagnant blood, and treat dysmenorrhea, tumors, diabetes, and wound in Asian countries [12,13,14,15]. Previous studies have reported several biologically active and structurally interesting constituents from *E. alatus* in the form of sesquiterpenes including sesquiterpene alkaloids, triterpenes, flavonoids, and phenolic compounds [16,17,18]. Among the constituents of *E. alatus*, a neolignan called abruslactone A was shown to exhibit anti-inflammatory effects in LPS-stimulated RAW-264.7 macrophages through the inhibition of the expression of iNOS [18].

As part of our ongoing research aimed for the discovery of bioactive constituents from Korean medicinal plants [19,20,21,22,23], we have explored the potential bioactive constituents of *E. alatus* [16,17,24,25]. Our previous studies have reported five new phenolic compounds with cytotoxicity and anti-neuroinflammatory activities [24], bioactive compounds that are antioxidants and/or *α*-glucosidase inhibitors [17], antiproliferative triterpenoids against A549, SK-OV-3, SK-MEL-2, and HCT-15 cell lines [25], and anti-inflammatory lignans [16] from the extracts of twigs of *E. alatus*. In this direction, we performed further phytochemical analysis of the methanolic (MeOH) extract of the twigs of *E. alatus* that led to the isolation of a sterol. This compound was identified as (3*β*,16*α*)-3,16-dihydroxypregn-5-en-20-one (**1**) with 1D and 2D nuclear magnetic resonance (NMR) spectroscopy and high-resolution electrospray ionization mass spectrometry (HR-ESIMS). The stereochemistry of **1** was established with nuclear Overhauser effect spectroscopy (NOESY) analysis and electronic circular dichroism (ECD) data. To the best of our knowledge, the isolation of compound **1** from nature, as well as its complete and revised assignment of NMR data are first reported herein. Compound **1** was evaluated for its inhibitory effect on NO production in LPS-activated RAW 264.7 macrophages based on its structural similarity with hydrocortisone, a well-known steroidal anti-inflammatory drug. In this study, we report the isolation, structural elucidation, and NMR assignment of compound **1** as well as its anti-inflammatory effect and the underlying mechanism of action.

## 2. Results and Discussion

### 2.1. Isolation and Identification of (3β,16α)-3,16-Dihydroxypregn-5-en-20-one

**Figure 1:**
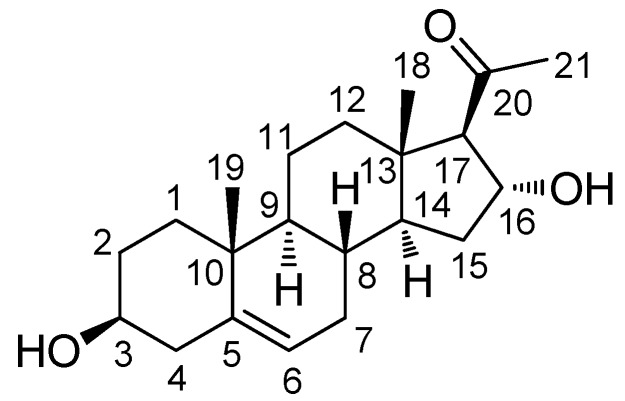
Structure of (3*β*,16*α*)-3,16-dihydroxypregn-5-en-20-one (**1**).

The MeOH extract of *E. alatus* twigs was fractionated using the solvent-partitioning method to obtain three soluble fractions (*n*-hexane-, CHCl_3_-, and *n*-BuOH-soluble fractions). Phytochemical analysis of the CHCl_3_-soluble fraction with repeated CC and semi-preparative HPLC monitored with LC/MS/UV-based analysis resulted in the isolation of one sterol (**1**; Figure 1).

**Figure 2:**
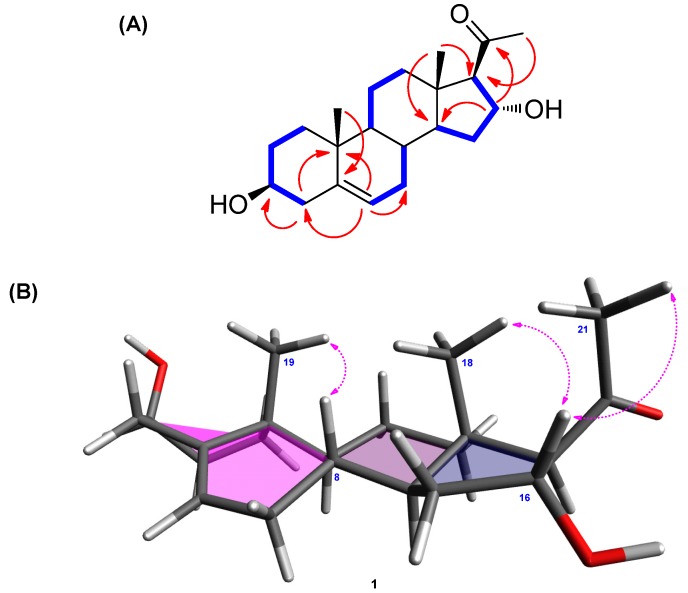
(**A**) ^1^H-^1^H COSY (bold lines) and key HMBC (arrows) correlations of **1**. (**B**) Key NOESY (arrows) correlations of **1**.

Compound **1** was obtained as a white powder. The molecular formula of **1** was determined to be C_21_H_32_O_3_ from the molecular ion peak [M + H]^+^ at *m/z* 333.2430 (calcd. for C_21_H_33_O_3_, 333.2430) in positive-ion HR-ESIMS (Figure S1). The ^1^H NMR spectrum (Table 1 and Figure S2) revealed the presence of signals corresponding to three tertiary methyl groups, including an acetyl group (*δ*_H_ 2.16 [3H, s, H_3_-21]), a deshielded methine (*δ*_H_ 2.55 [1H, d, *J* = 6.5 Hz, H-17]), two oxygenated methines (*δ*_H_ 3.40 [1H, m, H-3] and 4.72 [1H, m, H-16]), and an olefinic methine (*δ*_H_ 5.35 [1H, m, H-6]). The ^13^C NMR spectrum (Table 1 and Figure S3) showed 21 carbon signals, including three methyl groups (*δ*_C_ 13.3, 18.4, and 30.7) and four methines (*δ*_C_ 70.9, 71.4, 73.2, and 120.7), which were classified using heteronuclear single quantum coherence (HSQC) analysis (Figure S6), as well as four quaternary carbons, including an uncharacterized carbon (*δ*_C_ 140.9) and a carbonyl group (*δ*_C_ 209.3). Based on 2D-NMR data (^1^H-^1^H correlation spectroscopy [^1^H-^1^H COSY] (Figure S4) and heteronuclear multiple bond correlation [HMBC]) (Figure S7), compound **1** was identified as a steroid with an acetyl group at C-17 instead of a usual carbon chain (Figure 2A). The location of this acetyl group was confirmed from the HMBC of an acetyl group (*δ*_H_ 2.16) and C-17 (*δ*_C_ 73.2). The presence of one double bond was deduced from the corresponding ^1^H and ^13^C-NMR data (*δ*_H_ 5.35 [H-6] and *δ*_C_ 120.7 [C-6] and 140.9 [C-5]), and its location was confirmed from HMBC spectra, which revealed the correlations between H-6 (*δ*_H_ 5.35) and C-4 (*δ*_C_ 41.5), C-7 (*δ*_C_ 31.3), and C-10 (*δ*_C_ 36.3; Figure 2A). The existence of a hydroxyl group at C-3 was also confirmed with ^1^H-^1^H COSY correlations of H-3 [*δ*_H_ 3.40] and the neighboring protons, H-2 (*δ*_H_ 1.48, 1.80) and H-4 (*δ*_H_ 2.24). Furthermore, the presence of a hydroxyl group at C-16 was confirmed from the HMBC between H-16 (*δ*_H_ 4.72) and C-17 (*δ*_C_ 73.2), C-20 (*δ*_C_ 209.3), and C-14 (*δ*_C_ 54.3). The complete gross structure of **1** was further confirmed from the cross-peaks in ^1^H-^1^H COSY and HMBC spectra (Figure 2A).

The *β*-OH at C-3 was determined from the comparison between the ^1^H and ^13^C-NMR values of C-3 and the reported data of similar steroidal skeleton. As indicated in previous studies, the C-3 of steroids with *α*-OH moiety shows a slightly shielded chemical shift (*δ*_C_ 66.5 [26] and *δ*_C_ 67.1 [27]) in ^13^C-NMR data, whereas that of steroids with *β*-OH was reported to have deshielded values (*δ*_C_ 70.9 and *δ*_C_ 70.7) [26]. The difference between 3*β*-H and 3*α*-H at C-3 was clearly observed in previous studies [26,27], wherein the NMR signals of 3*β*-H appeared at around *δ*_H_ 3.90–4.05 and those of 3*α*-H appeared at a relatively shielded region between *δ*_H_ 3.50 and 3.65. In line with these evidences, the hydroxyl group at C-3 of **1** was confirmed to be *β*-oriented, as compound **1** had NMR values of *δ*_C_ 70.9 and *δ*_H_ 3.40. The remaining stereochemistry of **1** was established from the analysis of NOESY data (Figure S5), revealing the correlations between H-16/H-18 and H-16/H-21 as well as H-8/H-19 (Figure 2B). The stereochemistry of **1** was also determined from the ECD spectrum of **1** that exhibited a strong positive cotton effect at 288 nm, consistent with the results of a previous report [28]. Based on the above spectroscopic analysis, the complete structure of **1** was confirmed to be (3*β*,16*α*)-3,16-dihydroxypregn-5-en-20-one, which was reported in 2005 by Salvador et al. [29]. However, the structural assignment was not completely reported. The comparison of our data with the previously reported values [29] showed that the ^13^C-NMR values of C-3 and C-17 needed to be revised. According to Salvador et al. [29], the assigned values for C-3 and C-17 were reported to be *δ*_C_ 73.7 and *δ*_C_ 71.7, respectively; however, our analysis of 2D-NMR correlations clearly revealed that the values should be corrected as C-3 (*δ* 70.9) and C-17 (*δ* 73.2). To the best of our knowledge, the complete and revised assignment of NMR data for **1** was reported here for the first time.

### 2.2. Effect of Compound **1** on NO Production

**Figure 3:**
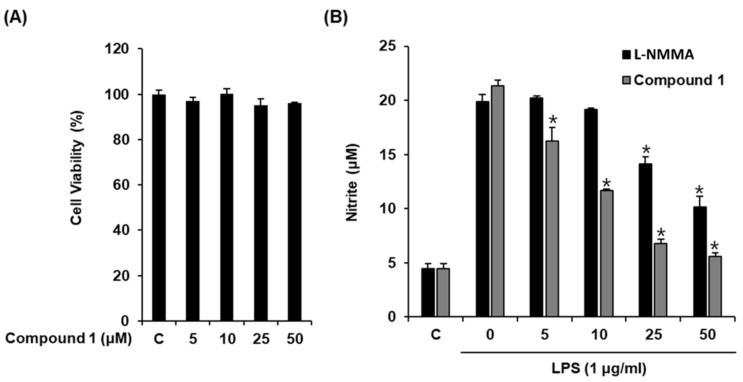
Effect of compound **1** on the lipopolysaccharide (LPS)-induced NO production in RAW 264.7 mouse macrophages. (**A**) The viability of RAW 264.7 cells incubated with compound **1** for 24 h was measured using an MTT assay. (**B**) The effect of compound **1** and l-NMMA as a positive control in LPS-treated RAW 264.7 macrophages was detected using the Griess reagent (mean ± SD, * *p* < 0.05 as compared to the LPS-treated group).

Macrophages are the main effector cells responsible for the innate immune response. After stimulation with LPS, macrophages release NO [30], a signaling molecule involved in inflammatory processes [31]. Therefore, NO production by LPS-activated RAW 264.7 macrophages serves as the measure of the anti-inflammatory effects of natural products. Considering that the structure of compound **1** resembles that of hydrocortisone, a well-known steroidal anti-inflammatory drug, we evaluated its inhibitory effect by determining NO production from LPS-activated RAW 264.7 macrophages. As shown in Figure 3, compound **1** significantly inhibited NO production at an IC_50_ value of 12.54 ± 0.05 μM (Figure 3B) without causing any significant cytotoxicity against RAW 264.7 cells after 24 h (Figure 3A). l-NMMA used as the positive control inhibited NO production with an IC_50_ value of 37.49 ± 0.41 μM (Figure 3B). Thus, compound **1** was more efficacious than the positive control in reducing NO production from LPS-activated RAW 264.7 macrophages. Thus, we sought to investigate the mechanism underlying its inhibitory effect.

### 2.3. Compound **1** Downregulates MAPKs (JNK, ERK, and p38) in LPS-Stimulated RAW 264.7 Mouse Macrophages

**Figure 4:**
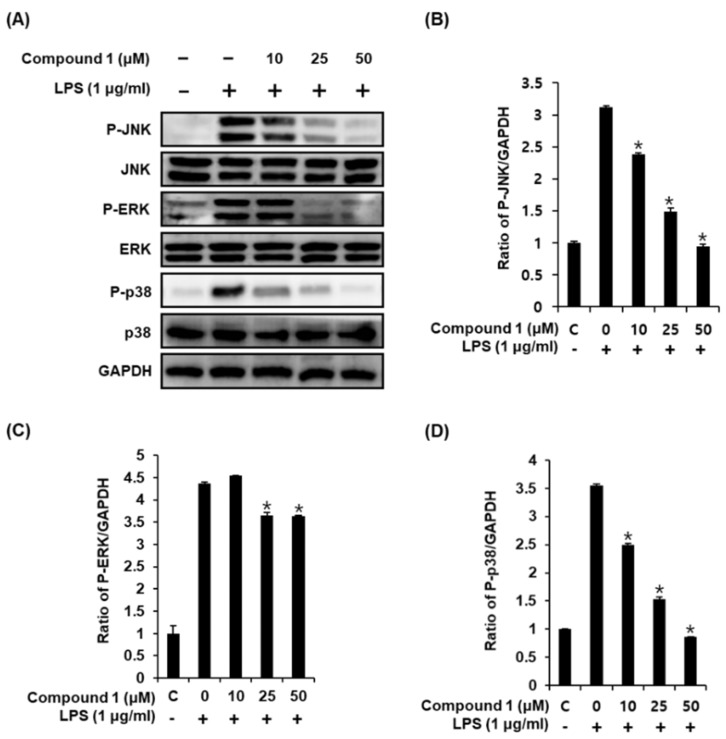
Effects of compound **1** on the LPS-induced expression of MAPKs (JNK, ERK, and p38) in RAW 264.7 mouse macrophages. (**A**) Representative western blots for JNK, ERK, p38, and GAPDH protein expression. (**B**) Quantitative graph for P-JNK. (**C**) Quantitative graph for P-ERK. (**D**) Quantitative graph for P-p38 (mean ± SD, * *p* < 0.05 as compared with the LPS-treated group).

Activation of LPS results in an increase in the phosphorylation of MAPKs, which comprises three subtypes, including JNK, ERK, and p38, in RAW 264.7 macrophages [32]. Upon phosphorylation of MAPKs, the transcription factors present in the cytoplasm or nucleus undergo phosphorylation and activation, consequently leading to the expression of proinflammatory mediators [33]. Western blot analysis was used to confirm whether the anti-inflammatory effect of compound **1** was related to MAPK pathways. As a result, we found that compound **1** (25, 50, and 100 μM) could effectively suppress the LPS-induced phosphorylation of JNK, ERK, and p38 at the protein level in a dose-dependent manner (Figure 4). These results suggest that compound **1** inhibits NO production by downregulating the expression of MAPKs (JNK, ERK, and p38) in LPS-stimulated RAW 264.7 mouse macrophages.

### 2.4. Compound **1** Downregulates IKKα/β, I-κBα, and NF-κB p65 in LPS-Stimulated RAW 264.7 Mouse Macrophages

**Figure 5:**
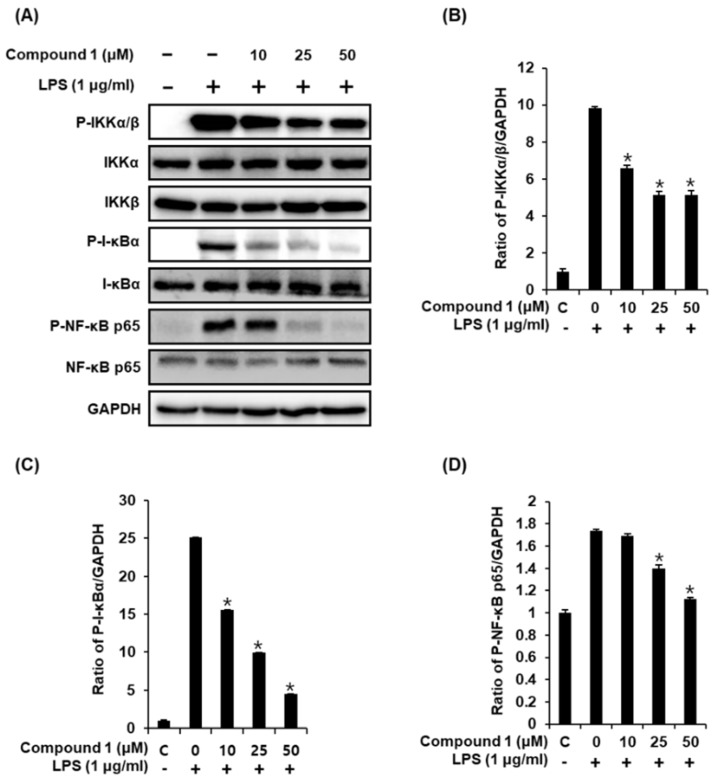
Effects of compound **1** on the LPS-induced expression of IKKα/β, I-κBα, and NF-κB p65 proteins in RAW 264.7 mouse macrophages. (**A**) Representative western blots for IKKα/β, I-κBα, NF-κB p65, and GAPDH protein expression. (**B**) Quantitative graph for P-IKKα/β. (**C**) Quantitative graph for P-I-κBα. (**D**) Quantitative graph for P-NF-κB p65 (mean ± SD, * *p* < 0.05 as compared with the LPS-treated group).

Following stimulation of macrophages with LPS, the phosphorylation of the IKK complex (IKKα and IKKβ) results in the phosphorylation and degradation of IκBα, thereby promoting the nuclear translocation of NF-κB [34]. The inducible transcription factor NF-κB subsequently activates the LPS-induced inflammatory gene expression in macrophages [35]. Therefore, we performed western blot analysis to determine whether compound **1** inhibits the activation of IKKα/β, I-κBα, and NF-κB p65 in LPS-stimulated RAW 264.7 mouse macrophages. As shown in Figure 5, the LPS-induced phosphorylation of IKKα/β, I-κBα, and NF-κB p65 was significantly reduced after treatment with compound **1**. These results suggest that compound **1** inhibited NF-κB p65 activation and IKKα/β and I-κBα functions in LPS-stimulated RAW 264.7 cells.

### 2.5. Compound **1** Downregulates iNOS and COX-2 Expression in LPS-Stimulated RAW 264.7 Mouse Macrophages

**Figure 6:**
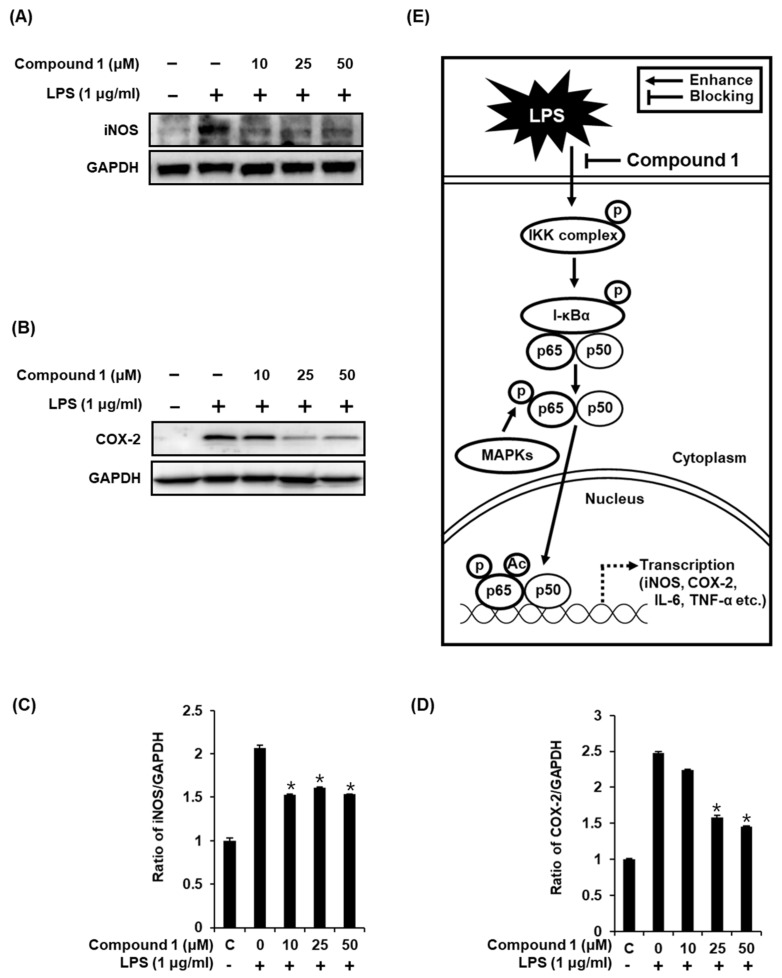
Effects of compound **1** on the LPS-induced expression of iNOS and COX-2 in RAW 264.7 mouse macrophages. (**A**) Representative western blots for iNOS and GAPDH protein expression. (**B**) Representative western blots for COX-2 and GAPDH protein expressions. (**C**) Quantitative graph for iNOS (mean ± SD, * *p* < 0.05 as compared with the LPS-treated group). (**D**) Quantitative graph for COX-2 (mean ± SD, * *p* < 0.05 as compared with the LPS-treated group). (**E**) Schematic model showing the role of compound **1** in inflammatory signaling pathways.

As previously reported, the increase in the nuclear expression of NF-κB p65 after LPS stimulation results in the induction of iNOS and COX-2 [3,4], which directly contributes to NO synthesis under inflammatory condition [36]. In the present study, the treatment of RAW 264.7 macrophages with LPS resulted in the activation of iNOS and COX-2, while compound **1** ameliorated this effect (Figure 6A,B). As shown in Figure 6C, the ability of compound **1** to inhibit NO production in LPS-activated RAW 264.7 macrophages was mediated through the inhibition of activation of MAPKs, IKKα/β, I-κBα, NF-κB 65, iNOS, and COX-2 (Figure 6E). These results further support the effective inhibition of LPS-induced NO production achieved with compound **1** treatment.

## 3. Materials and Methods

### 3.1. General Experimental Procedures

Optical rotations were calculated using a Jasco P-1020 polarimeter (Jasco, Easton, MD, USA). IR spectra were recorded on Bruker IFS-66/S FT-IR spectrometer (Bruker, Billerica, MA, USA) and UV spectra were acquired on an Agilent 8453 UV-visible spectrophotometer (Agilent Technologies, Santa Clara, CA, USA). NMR spectra were obtained using Bruker AVANCE III 500 NMR spectrometer (Bruker, Billerica, MA, USA) operating at 500 MHz (^1^H); the chemical shifts were reported in ppm (*δ*). Preparative high-performance liquid chromatography (HPLC) was performed using a Waters 1525 Binary HPLC pump with a Waters 996 Photodiode Array Detector (Waters Corporation, Milford, CT, USA). Silica gel 60 (Merck, 230–400 mesh, Kenilworth, NJ, USA) and RP-C18 silica gel (Merck, 230–400 mesh) as well as Sephadex LH-20 (Pharmacia, Uppsala, Sweden) were used for column chromatography (CC). Semi-preparative HPLC was performed with a Shimadzu Prominence HPLC System equipped with an SPD-20A/20AV Series Prominence HPLC UV-Vis Detector (Shimadzu, Tokyo, Japan). LC/MS analyses were carried out with an Agilent 1200 Series HPLC system (Agilent Technologies, Santa Clara, CA, USA) equipped with a diode array detector and a 6130 Series ESI mass spectrometer with an analytical Kinetex (4.6 × 100 mm, 3.5 μm) HPLC column. Kieselgel 60 F_254_-precoated Al cards (0.2-mm thickness, Merck) were used for thin-layer chromatography (TLC) plates, and visualization was carried out under UV light at 254/365 nm wavelength by spraying anisaldehyde-H_2_SO_4_.

### 3.2. Plant Material

The twigs of *E. alatus* were collected from Chungju, Chungcheongbuk-do, Korea, in March 2010. Plant material samples were identified by one of the authors (Kim, K.H.). A voucher specimen (SKKU 2010-3) has been deposited in the herbarium of the School of Pharmacy, Sungkyunkwan University, Suwon, Korea.

### 3.3. Extraction and Isolation

The finely chopped twigs of *E. alatus* (5.2 kg) were subjected to extraction with 80% aqueous MeOH twice (each 20 L × 4 h) under reflux and then filtered. The filtrate was evaporated under the vacuum to obtain a crude MeOH extract (220.0 g), which was suspended in distilled water (3.6 L) and successively solvent-partitioned with hexane, chloroform (CHCl_3_), and *n*-BuOH to yield 31.0, 11.7, and 35.4 g of residue, respectively. The CHCl_3_-soluble fraction (11.7 g) was subjected to silica CC (SiO_2_; CHCl_3_/MeOH, 20:1, 10:1, 1:1) to obtain seven fractions (Fr. A1–A7). Fr. A1–A3 were consolidated on the basis of TLC analysis, and the combined fraction (2.3 g) was separated with reverse-phase CC using a gradient solvent system of MeOH/H_2_O (1:1, 4:1, and 1:0) to obtain 16 subfractions (Fr. B1–B16). Fr. B6 (150.0 mg) was applied onto a Sephadex LH-20 column using a solvent system of CH_2_Cl_2_/MeOH (1:1) to produce three fractions (Fr. B6.1–B6.3). Fr. B6.3 (48.0 mg) was purified with semi-preparative normal-phase HPLC using a solvent system of CH_2_Cl_2_/MeOH (30:1) to yield compound **1** (10.0 mg).

#### (3β,16α)-3,16-Dihydroxypregn-5-en-20-one (**1**):

White powder ${\left[\alpha \right]}_{D}^{25}$ -8.5 (*c* 0.09, MeOH); IR (KBr) *ν*_max_ 3405, 2946, 1670, 1033 cm^−1^; UV (MeOH) λ_max_ (log *ε*) 205 (2.8) nm; ECD (MeOH) λ_max_ (Δ*ε*) 288 (+2395.6) nm; ^1^H and ^13^C-NMR data, see Table 1; HR-ESIMS (positive-ion mode) *m/z* 333.2430 [M + H]^+^ (calcd. for C_21_H_33_O_3_, 333.2430).

### 3.4. Cell Culture

RAW 264.7 (American Type Culture Collection, Rockville, MD, USA), a mouse macrophages cell line, was cultured in Dulbecco’s modified Eagle’s medium (Cellgro, VA, USA) supplemented with 10% fetal bovine serum (FBS), 1% penicillin/streptomycin (Invitrogen Co., Waltham, MA, USA), and 4 mM l-glutamine at 37 °C in an atmosphere of 5% CO_2_.

### 3.5. Measurement of RAW 264.7 Cell Viability

RAW 264.7 cell viability was assessed with an Ez-Cytox cell viability detection kit (Daeil Lab Service Co., Seoul, Korea). Cells were seeded into a 96-well plate at a density of 3 × 10^4^ cells/well and treated with compound **1** for 24 h at 37 °C. Ez-Cytox solution was added to each well and the plate was incubated for 40 min at 37 °C. Absorbance at 450 nm wavelength was recorded using a PowerWave XS microplate reader (Bio-Tek Instruments, Winooski, VT, USA).

### 3.6. Measurement of NO Production in RAW 264.7 Cells

NO production in RAW 264.7 cells was assessed with the Griess reagent comprising 0.2% naphthylethylenediamine dihydrochloride (Sigma-Aldrich, St. Louis, MO, USA) and 2% sulfanilamide (Sigma-Aldrich, St. Louis, MO, USA) in 5% phosphoric acid (Sigma-Aldrich, St. Louis, MO, USA). Cells were seeded in a 96-well plate at a density of 3 × 10^4^ cells/well and pretreated with compound **1** or *N*^G^-methyl-l-arginine acetate salt (l-NMMA), a nitric oxide synthase inhibitor, as a positive control for 1 h. LPS (1 μg/mL) was added to each well and the plate was incubated for 40 min at 37 °C. Cell culture media were mixed with an equal volume of Griess reagent and absorbance at 540 nm was measured using PowerWave XS microplate reader.

### 3.7. Western Blot Analysis

Specific proteins were analyzed with epitope-specific primary antibodies to phospho-JNK, JNK, phospho-ERK, ERK, phospho-p38, p38, phospho-IKKα/β, IKKα (IκB kinase alpha), IKKβ (IκB kinase beta), phospho-I-κBα, I-κBα (inhibitor of kappa B alpha), phospho-NF-κB p65, NF-κB p65, iNOS, COX-2, glyceraldehyde 3-phosphate dehydrogenase (GAPDH), and horseradish peroxidase (HRP)-conjugated anti-rabbit antibodies (Cell Signaling, Danvers, MA, USA) using western blotting. Equal amounts of protein samples (20 μg total protein per lane) were separated by electrophoresis on a 10% sodium dodecyl sulfate-polyacrylamide gel and then transferred onto a polyvinylidene fluoride (PVDF) membrane. The PVDF membrane was incubated with epitope-specific primary antibodies overnight at 4 °C, followed by incubation with HRP-conjugated anti-rabbit antibodies for 1 h at room temperature. The bound antibodies were developed using enhanced chemiluminescence (ECL) Advance Western Blotting Detection Reagents (GE Healthcare, Little Chalfont, UK) and scanned with a FUSION Solo Chemiluminescence System (PEQLAB Biotechnologie GmbH, Germany).

### 3.8. Statistical Analysis

All the assays were performed in triplicates and repeated at least thrice. All data were presented as the average value and standard deviation (SD). Statistical significance was determined using the one-way analysis of variance (ANOVA) and multiple comparisons with a Bonferroni correction. A value of *p* less than 0.05 indicated statistical significance. All analyses were performed using SPSS Statistics ver. 19.0 (SPSS Inc., Chicago, IL, USA).

## 4. Conclusions

This study provides an experimental evidence of the potential role of (3*β*,16*α*)-3,16-dihydroxypregn-5-en-20-one in the management and treatment of inflammatory diseases. The compound (3*β*,16*α*)-3,16-dihydroxypregn-5-en-20-one (**1**) was isolated from the twigs of *E. alatus*, which have been used as a remedy for various diseases, including dysmenorrhea, tumors, diabetes, and wound. In addition, the isolation of compound **1** from natural sources and the complete and revised NMR data of **1** are reported for the first time. Based on the structural similarity between compound **1** and hydrocortisone, we found that compound **1** exhibited anti-inflammatory effects by inhibiting NO production in the LPS-activated RAW 264.7 macrophages. The inhibitory effect of **1** was mediated through the down regulation of the activation of MAPKs, IKKα/β, I-κBα, NF-κB p65, iNOS, and COX-2. These findings provide experimental evidence that compound **1** from *E. alatus* twigs could be an effective agent for the treatment of inflammatory diseases.

## Figures and Tables

**Table 1:** ^1^H and ^13^C-NMR data of compound **1** (CD_3_OD) and its reported value (CDCl_3_) [29].

Position	1		1 [29]	
	*δ*_H_ *^a^*	*δ*_C_ *^a^*	*δ*_H_	*δ*_C_
1	1.11 dd (13.5, 3.5), 1.88 dt (13.5, 3.5)	37.0		
2	1.48 m, 1.80 m	30.8		
3	3.40 m	70.9	3.48 m	73.7
4	2.24 m	41.5		
5		140.9		141.1
6	5.35 m	120.7	5.34 d (4.0)	121.3
7	1.64 m, 1.96 m	31.3		
8	2.20 m	31.3		
9	1.07 m	50.1		
10		36.3		
11	1.50 m, 1.69 m	20.4		
12	1.57 m *^b^*, 2.01 m	38.5		
13		44.7		
14	1.56 m *^b^*	54.3		
15	1.54 m *^b^*, 1.72 d (8.5)	35.1		
16	4.72 m	71.4	4.77 m	71.3
17	2.55 d (6.5)	73.2	2.57 d (6.5)	71.7
18	0.64 s	13.3	0.64 s	
19	1.02 s	18.4	1.01 s	
20		209.3		210.2
21	2.16 s	30.7	2.19 s	

*^a^* Assignments were based on HSQC, HMBC, and ^1^H-^1^H COSY experiments. *^b^* Overlapped.

## Supplementary Materials

HRESIMS and 1D and 2D NMR data of (3*β*,16*α*)-3,16-dihydroxypregn-5-en-20-one are available free of charge on the Internet.
