# Supplementary material for: (3β,16α)-3,16-Dihydroxypregn-5-en-20-one from the Twigs of Euonymus alatus (Thunb.) Sieb. Exerts Anti-Inflammatory Effects in LPS-Stimulated RAW-264.7 Macrophages
Source: Molecules. 2019 Oct 25;24(21):3848. doi: 10.3390/molecules24213848 (PMC6864714; doi:10.3390/molecules24213848)

Supplementary Materials

(3*β*,16*α*)-3,16-Dihydroxypregn-5-en-20-one from the Twigs of *Euonymus alatus* (Thunb.) Sieb. Exerts Anti-inflammatory Effects in LPS-stimulated RAW-264.7 Macrophages

Seulah Lee [1,†], Dahae Lee [1,†], Su Cheol Baek [1], Mun Seok Jo [1], Ki Sung Kang [2,*], and Ki Hyun Kim [1,*]

[1] School of Pharmacy, Sungkyunkwan University, Suwon 16419, Republic of Korea

[2] College of Korean Medicine, Gachon University, Seongnam 13120, Republic of Korea

* Corresponding author:

Ki Sung Kang, Tel.: +82-31-750-5402; E-mail: : kkang@gachon.ac.kr

Ki Hyun Kim, Tel: +82-31-290-7700; Fax: +82-31-290-7730; E-mail: khkim83@skku.edu

[†] These authors contributed equality to this work.

Supporting Information Contents:

Figure S1. HR-ESIMS data of **1**……S3
Figure S2. ¹H NMR spectrum of **1** (CD_3OD, 700 MHz)……S4
Figure S3. DEPT spectrum of **1** (CD_3OD, 175 MHz)……S5
Figure S4. ¹H-¹H COSY spectrum of **1** (CD_3OD)……S6
Figure S5. NOESY spectrum of **1** (CD_3OD)……S7
Figure S6. HSQC spectrum of **1** (CD_3OD)……S8
Figure S7. HMBC spectrum of **1** (CD_3OD)……S9

Figure S1. HR-ESIMS data of **1**.

Single Mass Analysis
Tolerance = 5.0 mDa / DBE: min = -1.5, max = 50.0
Element prediction: Off
Number of isotope peaks used for i-FIT = 3
Monoisotopic Mass, Even Electron Ions
63 formula(e) evaluated with 1 results within limits (up to 50 closest results for each mass)
Elements Used:

Mass	Calc. Mass	mDa	PPM	DBE	Formula	i-FIT	i-FIT Norm	Fit Conf %	C	H	O
333.2430	333.2430	0.0	0.0	5.5	C21 H33 O3	250.1	n/a	n/a	21	33	3

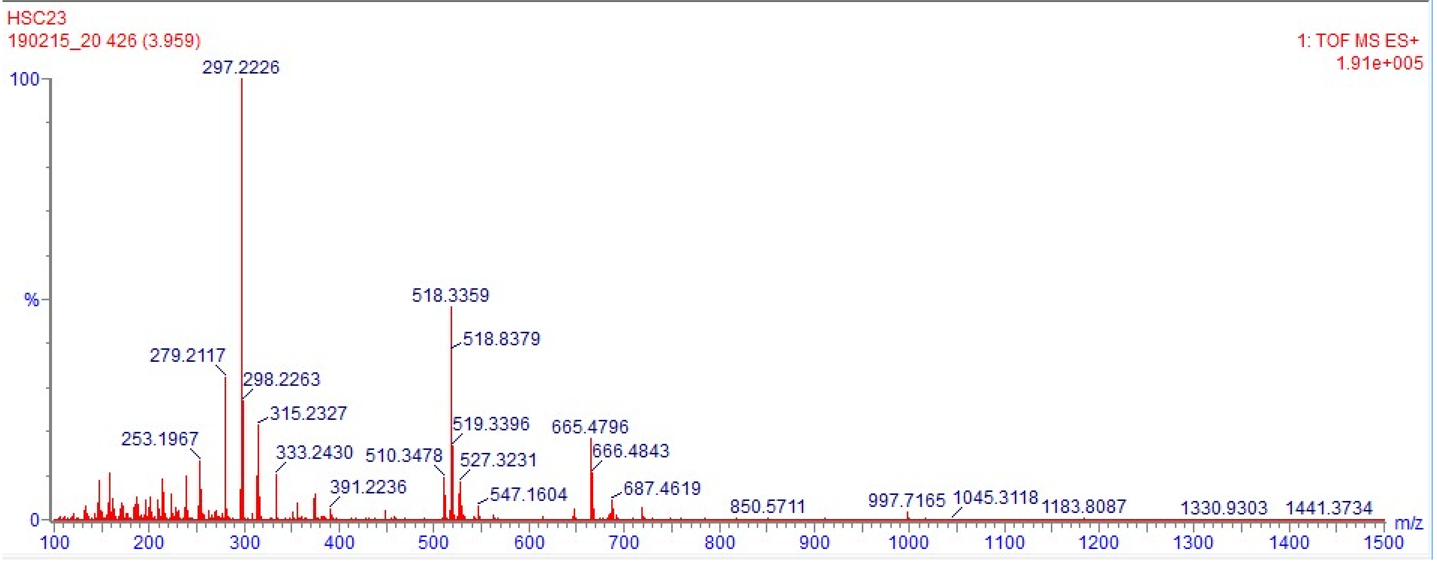

Figure S2. ^{1}H NMR spectrum of **1** (CD_3OD, 700 MHz).

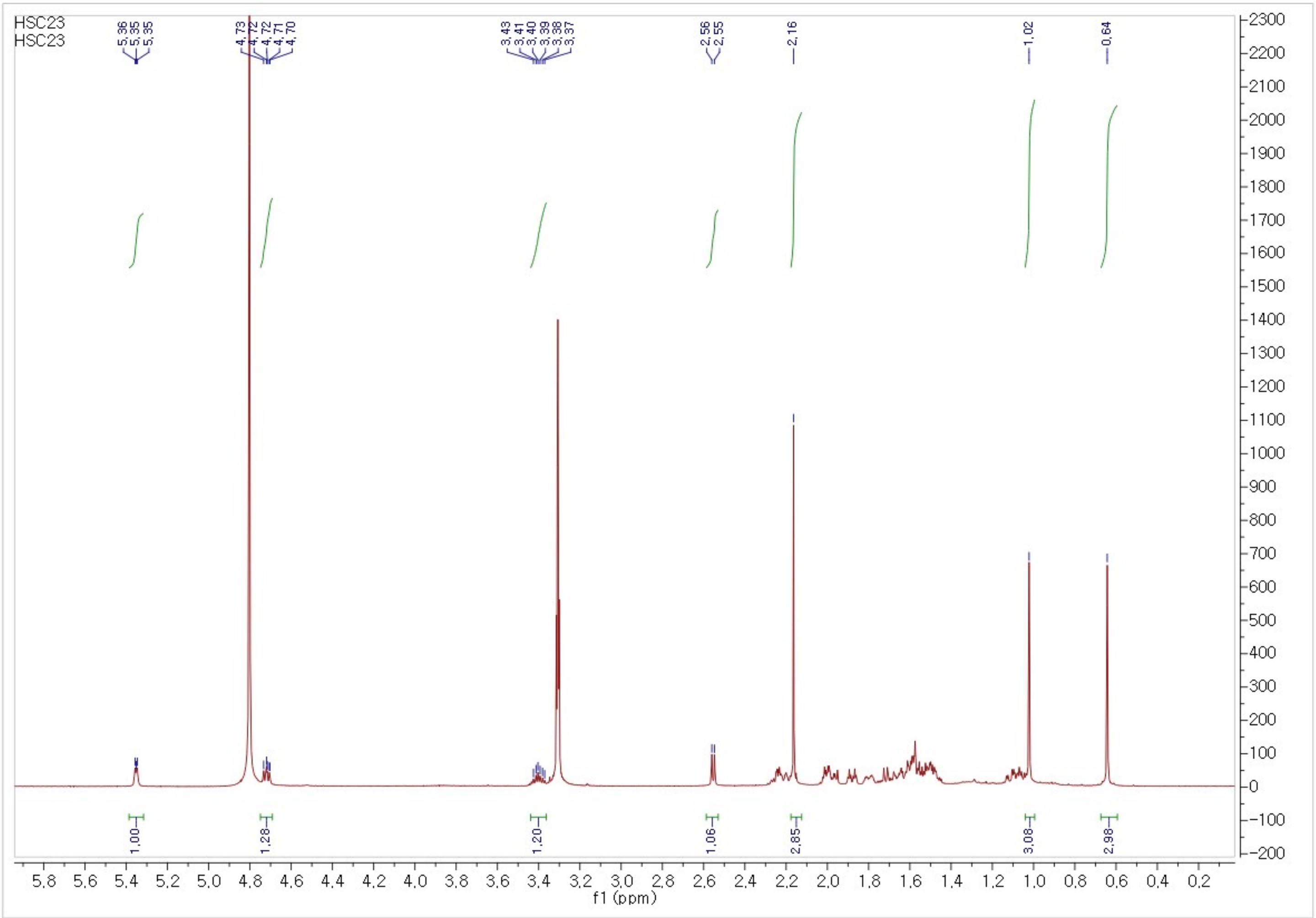

Figure S3. DEPT spectrum of **1** (CD_3OD, 175 Hz).

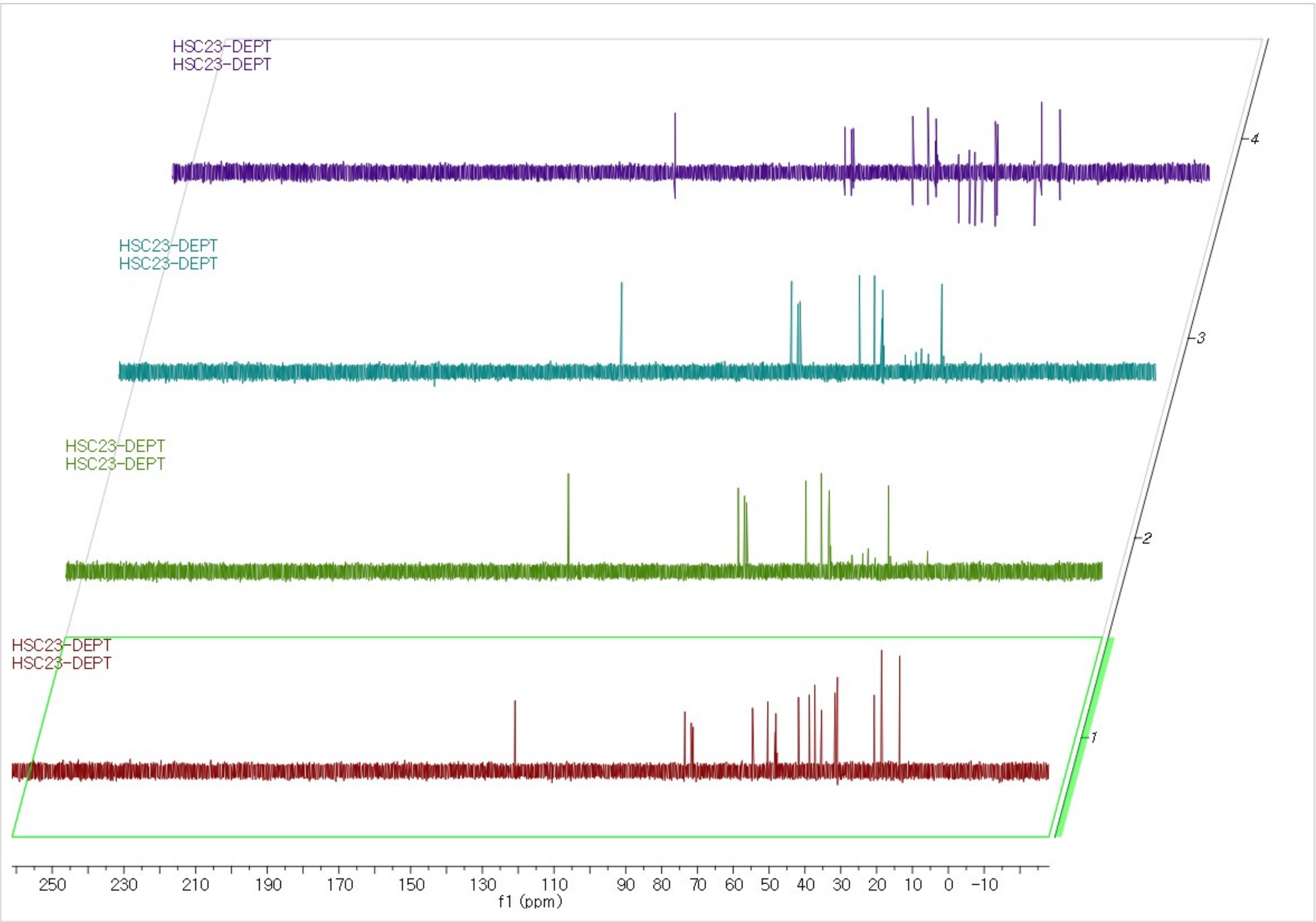

Figure S4. ^{1}H-^{1}H COSY spectrum of **1** (CD_3OD).

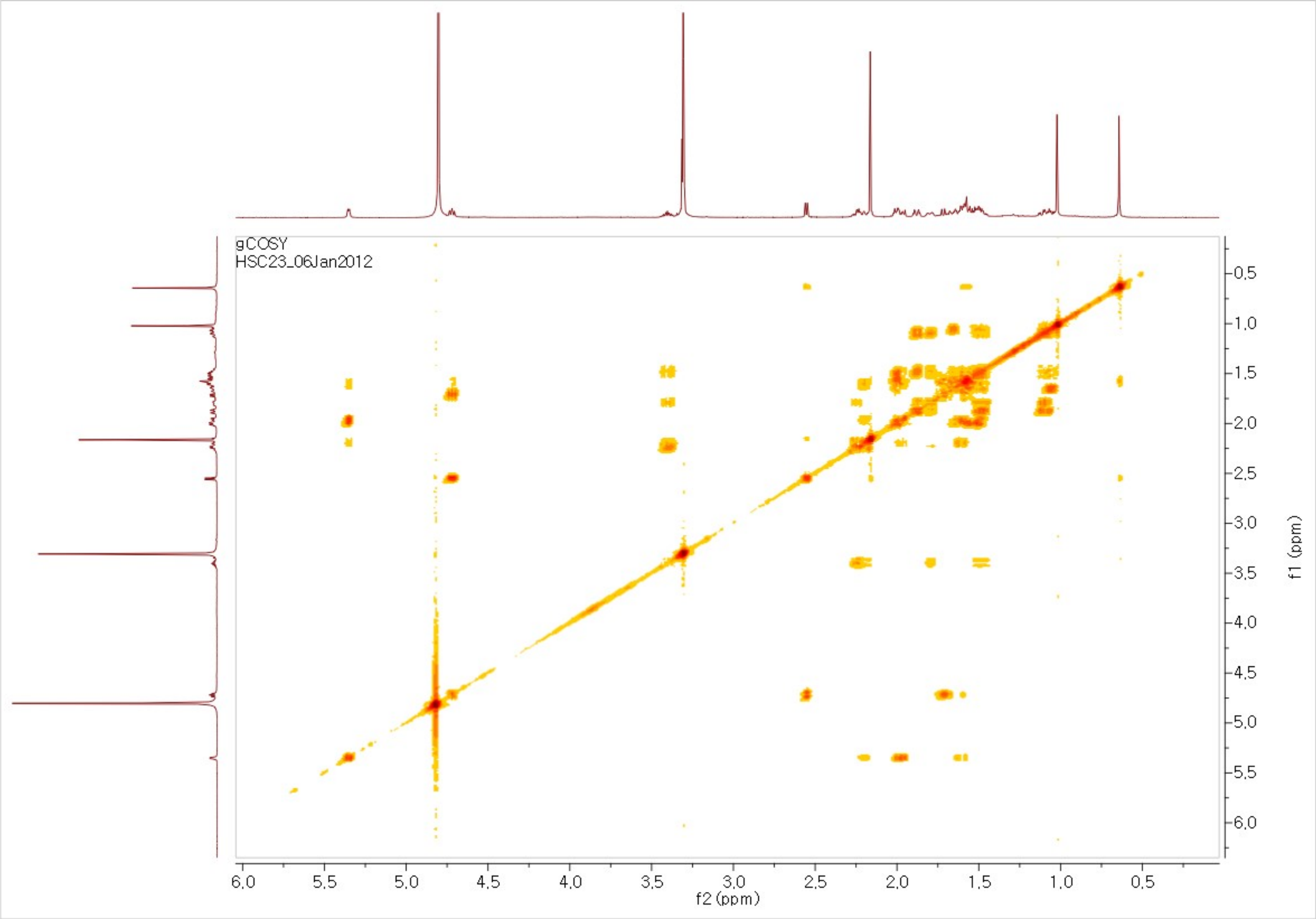

Figure S5. NOESY spectrum of **1** (CD_3OD).

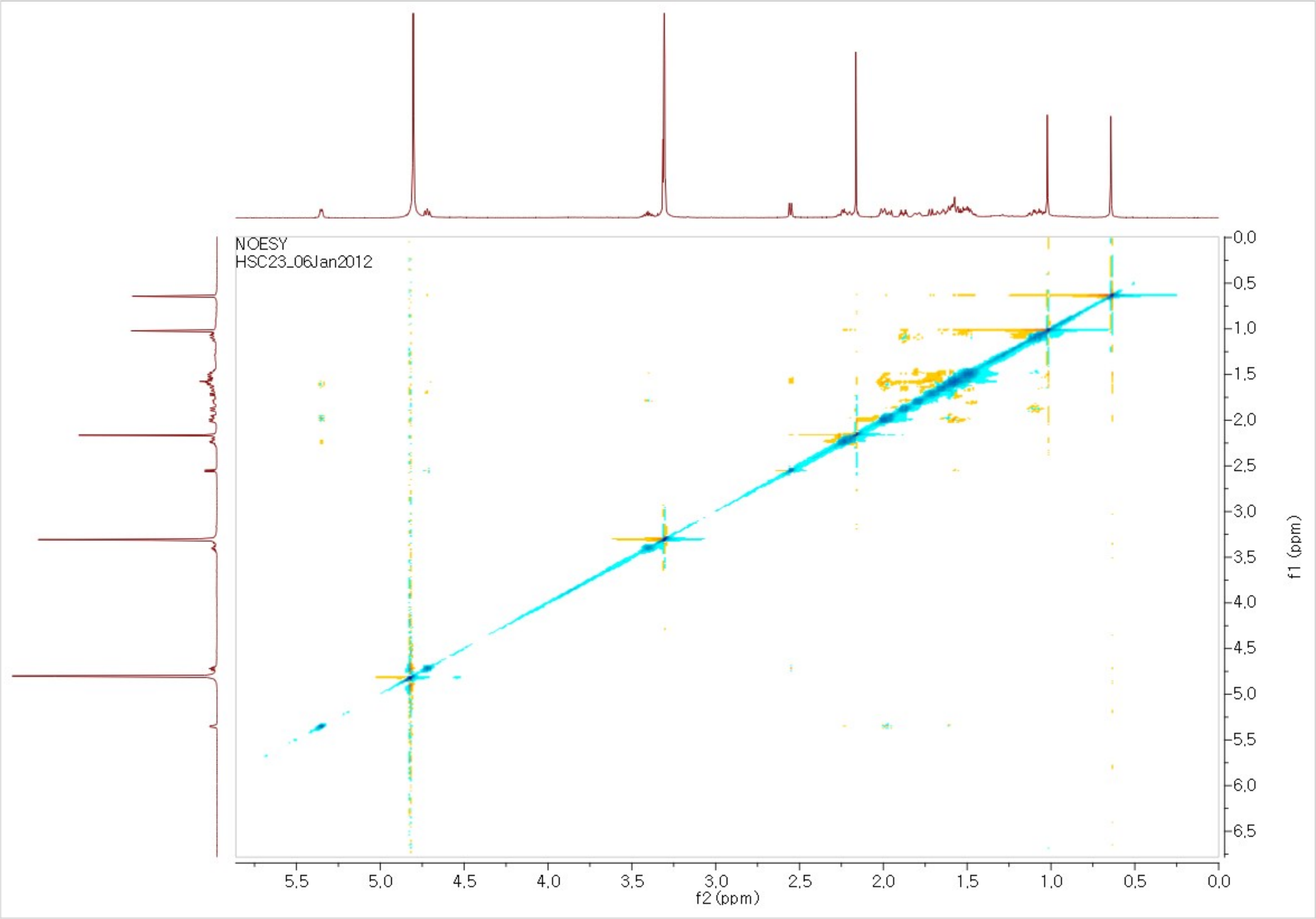

Figure S6. HSQC spectrum of **1** (CD_3OD).

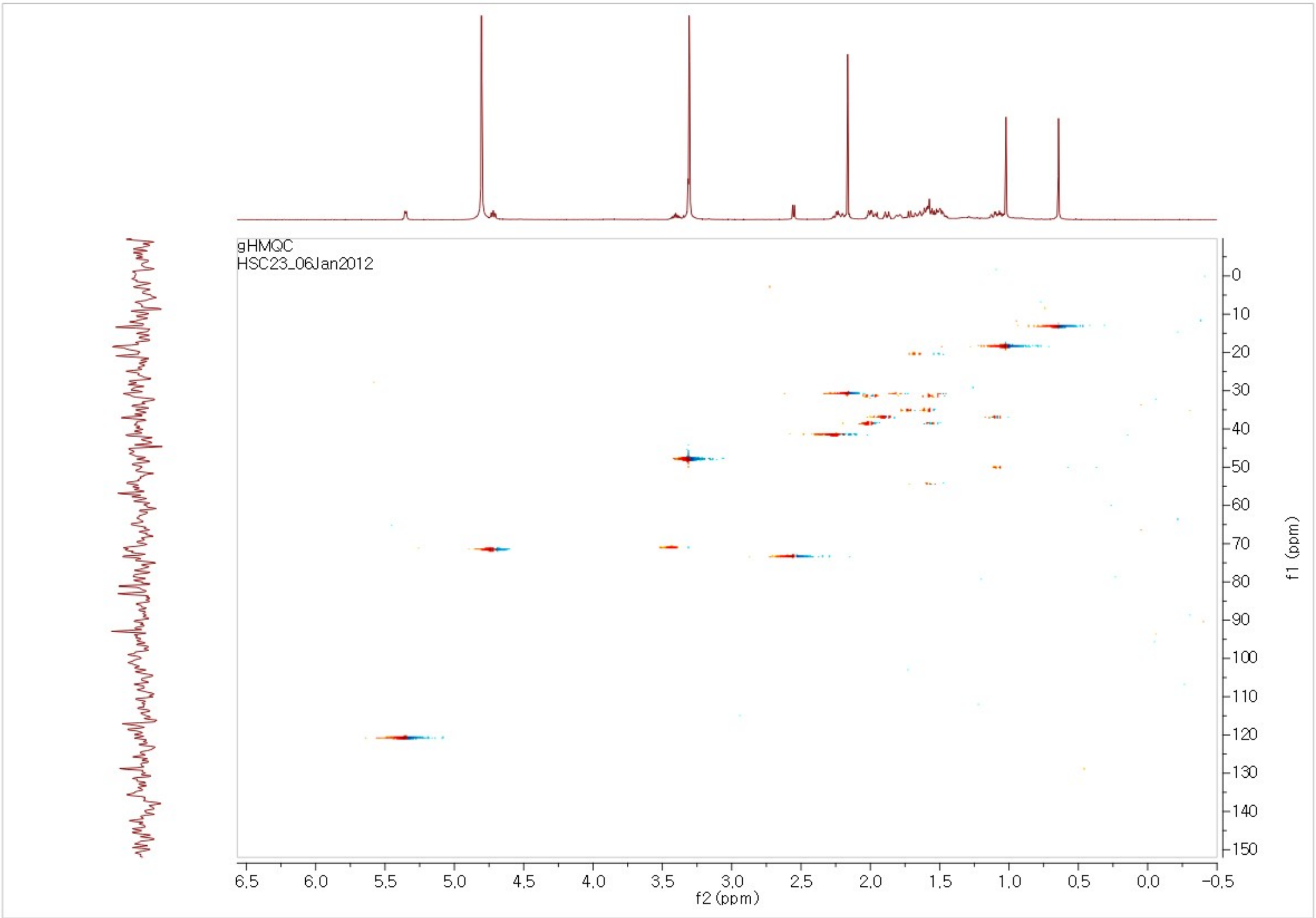

Figure S7. HMBC spectrum of **1** (CD_3OD).

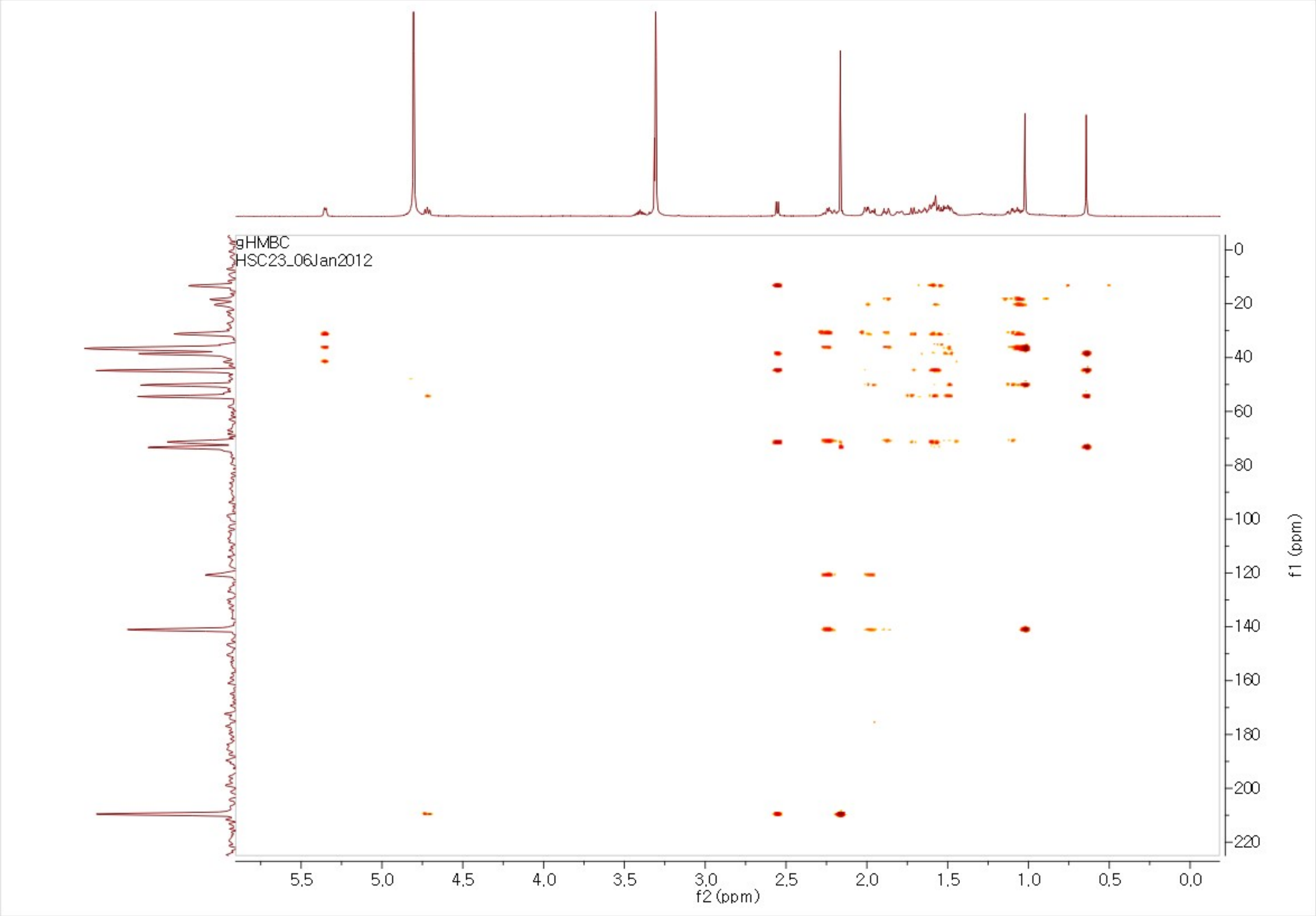

Supplement: Supplementary file 1 [file molecules-24-03848-s001.pdf]
